# Serum Glucose and Fructosamine in Relation to Risk of Cancer

**DOI:** 10.1371/journal.pone.0054944

**Published:** 2013-01-25

**Authors:** Wahyu Wulaningsih, Lars Holmberg, Hans Garmo, Björn Zethelius, Annette Wigertz, Paul Carroll, Mats Lambe, Niklas Hammar, Göran Walldius, Ingmar Jungner, Mieke Van Hemelrijck

**Affiliations:** 1 King’s College London, School of Medicine, Division of Cancer Studies, Cancer Epidemiology Group, London, United Kingdom; 2 Department of Surgical Sciences, Uppsala University Hospital, Uppsala, Sweden; 3 Regional Cancer Centre, Uppsala, Sweden; 4 Department of Public Health/Geriatrics, Uppsala University, Uppsala, Sweden; 5 Medical Products Agency/Epidemiology, Uppsala, Sweden; 6 Department of Endocrinology, Guy’s & St Thomas’ NHS Foundation Trust, St. Thomas’ Hospital, London, United Kingdom; 7 Department of Medical Epidemiology and Biostatistics, Karolinska Institutet, Stockholm, Sweden; 8 Department of Epidemiology, Insitute of Environmental Medicine, Karolinska Institutet, Stockholm, Sweden; 9 AstraZeneca Sverige, Södertalje, Sweden; 10 Department of Epidemiology, Institute of Environmental Medicine, Karolinska Institutet, Stockholm, Sweden; 11 Department of Medicine, Clinical Epidemiological Unit, Karolinska Institutet and CALAB Research, Stockholm, Sweden; University of Pecs Medical School, Hungary

## Abstract

**Background:**

Impaired glucose metabolism has been linked with increased cancer risk, but the association between serum glucose and cancer risk remains unclear. We used repeated measurements of glucose and fructosamine to get more insight into the association between the glucose metabolism and risk of cancer.

**Methods:**

We selected 11,998 persons (>20 years old) with four prospectively collected serum glucose and fructosamine measurements from the Apolipoprotein Mortality Risk (AMORIS) study. Multivariate Cox proportional hazards regression was used to assess standardized log of overall mean glucose and fructosamine in relation to cancer risk. Similar analyses were performed for tertiles of glucose and fructosamine and for different types of cancer.

**Results:**

A positive trend was observed between standardized log overall mean glucose and overall cancer risk (HR = 1.08; 95% CI: 1.02–1.14). Including standardized log fructosamine in the model resulted in a stronger association between glucose and cancer risk and aninverse association between fructosamine and cancer risk (HR = 1.17; 95% CI: 1.08–1.26 and HR: 0.89; 95% CI: 0.82–0.96, respectively). Cancer risks were highest among those in the highest tertile of glucose and lowest tertile of fructosamine. Similar findings were observed for prostate, lung, and colorectal cancer while none observed for breast cancer.

**Conclusion:**

The contrasting effect between glucose, fructosamine, and cancer risk suggests the existence of distinct groups among those with impaired glucose metabolism, resulting in different cancer risks based on individual metabolic profiles. Further studies are needed to clarify whether glucose is a proxy of other lifestyle-related or metabolic factors.

## Introduction

Impaired glucose metabolism is one of the most common lifestyle-related disorders and has been linked to various chronic diseases including cancer [1]–[4]. It is unclear whether this association is merely caused by the many shared risk factors between impaired glucose metabolism and cancer or whether derangements in the glucose metabolism per se increase the risk of cancer [5]. Biological evidence suggests that elevated glucose levels, both directly through the activation of Receptor for Advanced Glycation End-products (RAGE) axis and indirectly through the mitogenic effect of insulin, may promote sequential events leading to cancer development [6]–[8]. In epidemiological studies, serum glucose was reported to be related to risk of incident cancer in large Korean and European cohorts [9], [10]. Most studies defined serum glucose captured in a single measurement, which may be prone to within-person variability [11]. To date, only two studies used repeated measurements of glucose when assessing risk of colorectal and breast cancer [12], [13].

Besides serum glucose, other markers of the glucose metabolism have been used to monitor diabetic patients. Fructosamine, which refers to glycated serum proteins, reflects the average glucose levels for the previous 10–14 days and thus serves as a more stable indicator of short-term glycaemic status compared to serum glucose [14]. In contrast to glucose, little evidence has been found for a link between fructosamine and cancer risk [15], [16]. We aimed to investigate the association between impaired glucose metabolism and cancer risk using repeated measurements of both serum glucose and fructosamine in a prospective cohort of 11,998 persons.

## Methods

### Study Population and Data Collection

The Apolipoprotein MOrtality RISk (AMORIS) study contains 351,487 men and 338,101 women recruited during the period 1985 to 1996, mainly from the greater Stockholm area [17], [18]. Blood samples of the participants were sequentially sent to the Central Automation Laboratory (CALAB) located in Stockholm, Sweden. This major laboratory has served more than 3,000 physicians in the Swedish healthcare system, and was acknowledged for Good Laboratory Practice and internationally accredited in clinical chemistry, hematology, immunology, and microbiology [19], [20]. Individuals recruited were either healthy and having a laboratory testing as a part of general check-up, or outpatients referred for laboratory testing. None of the participants were inpatients at the time the samples were analyzed. Apart from information on blood testing, no clinical data were available. Using the Swedish 10-digit personal identity number, the CALAB database was linked to several Swedish national registries including the Swedish National Cancer Register, the National Patient Register, the Cause of Death Register, the consecutive Swedish Censuses during 1970–1990, and the Population Register, providing information on cancer diagnosis, co-morbidities, vital status, socioeconomic status (SES), and emigration. This linkage is called AMORIS and complies with the Declaration of Helsinki and was approved by the Ethics Review Board of the Karolinska Institute.

From the AMORIS population, we selected all participants aged 20 or older who had four repeated measurements of glucose and fructosamine (n = 11,998). To define a repeated measurement we used the following criteria: the interval between each measurement had to be at least 9 months but maximum 15 months, and the interval between the first and the fourth measurements could not exceed 45 months. These intervals were chosen to capture annual changes in glycaemic status. All participants were free from cancer at time of entry and none were diagnosed with cancer or died within three months after study entry. The variables regarded as exposures in this study were serum glucose (mmol/L) and fructosamine (mmol/L). Standardized logarithm of overall mean glucose and fructosamine were calculated to observe the effect of small changes in glucose and fructosamine levels. Since fructosamine levels may be affected by serum albumin, we also assessed corrected fructosamine (c-fructosamine) using the following formula: (fructosamine (mmol/L)/albumin (g/L))×100 [21]. Furthermore, we used tertiles of overall mean glucose from the general population (<4.67, 4.67–5.13, ≥5.13 mmol/L) in combination with tertiles of overall mean fructosamine (<2.03, 2.03–2.17, ≥2.17 mmol/L).

The following data were also collected from the CALAB database: total cholesterol (mmol/L), triglycerides (mmol/L), and age at baseline. Height (cm) and weight (kg) were recorded for 2,828 (24%) subjects and used to determine body mass index (BMI). The mean total cholesterol and triglycerides levels recorded at the same time as the four glucose and fructosamine measurements were also calculated and used in the analysis. All laboratory examinations were performed with automated and calibrated instruments in CALAB laboratory [19]. Glucose was measured enzymatically with a glucose oxidase/peroxidase method. Fructosamine concentration was determined with colorimetric method on the basis of nitroblue tetrazolium reduction, whereas albumin was measured with a colorimetric method based on its binding with bromocresol green. Total cholesterol and triglycerides were determined enzymatically with oxidase-peroxidase assay.

Information regarding cancer diagnosis was obtained from Swedish National Cancer Register, using ICD-7 codes to identify overall (140–205), prostate (177), breast (170), colorectal (153, 154), and lung cancer (162). From the Population and Housing Census, information about socioeconomic status (SES) was also collected. SES is based on occupational groups and classifies all gainfully employed subjects as manual workers and nonmanual workers, which are referred to below as blue collar and white collar workers [22]. Information on age at first child birth and parity was obtained from the Swedish Multi-Generation Register and was specifically used in the analyses for breast cancer. From the National Patient Register we took information about hospitalization for diabetes, cardiovascular and lung disease. The latter was used as a proxy for smoking.

### Data Analysis

Multivariate Cox proportional hazards regression was used to study the association between glucose, fructosamine, and cancer. We assessed the standardized log of overall mean serum glucose and fructosamine in relation to overall cancer risk using four different models. In the first model, we evaluated the association between standardized log of overall mean glucose or fructosamine and risk of cancer,while adjusting for age. Next, we assessed cancer risk while including standardized log of overall mean glucose and fructosamine in the same model and adjusting for age. Additional adjustment were subsequently conducted for other potential confounding factors including gender, SES, fasting status, history of cardiovascular and lung disease, history of diabetes, serum albumin, triglycerides and total cholesterol levels. Additionally, we performed a sensitivity analysis for persons aged 50 and older in the study population. To further minimize confounding effects by history of diabetes, we repeated the above analyses among all non-diabetic persons (n = 10,743). These were defined as those with glucose <7.0 mmol/L at all measurements and without any registered hospital discharge diagnosis of diabetes prior to the fourth measurement. As obesity has also been linked to both impaired glucose metabolism and cancer [23], we also repeated the above analyses among those with baseline BMI values (N = 2,828), while adjusting for BMI. Furthermore, we assessed the risk of the most common individual cancers in the study population, i.e. prostate, breast, colorectal, and lung cancer, using the methods described above. To identify any interaction between glucose and fructosamine, we performed likelihood ratio test for all the above models. Finally, we evaluated the pattern of glucose and fructosamine levels in relation to cancer risk by looking at tertiles of overall mean glucose and those of fructosamine. We also repeated this analysis for risk of prostate, breast, colorectal, and lung cancer. To evaluate selection bias we performed the above analyses in another AMORIS subcohort based on all persons aged >20 years with at least one measurement of glucose and fructosamine (n = 402,026). Follow-up time was defined as time from the first measurement (for the single measurement) or fourth measurement (for repeated measurements) until date of cancer diagnosis, death, emigration, or end of follow-up (31^st^ December 2002), whichever occurred first. All analyses were conducted with Statistical Analysis Systems (SAS) release 9.1.3 (SAS Institute, Cary, NC).

## Results

During a mean follow-up of 9.4 years, 1,021 participants (8.51%) developed cancer. More cancers were observed in men (57.79%). An overview of the study population is given in Table 1. Using an analysis of variance for the four repeated measurements of glucose and fructosamine, we found no statistically significant changes in glucose and fructosamine levels over time (results not shown).

**Table 1:** Characteristics of study participants based on cancer status.

		N (%)	
		Cancer(N = 1021)	No Cancer(N = 10977)
**Age**	Mean (SD)	62.82 (11.84)	52.88 (14.90)
	20–30	7 (0.69)	792 (7.22)
	30–40	24 (2.35)	1273 (11.60)
	40–50	95 (9.30)	2588 (23.58)
	50–60	274 (26.84)	2768 (25.22)
	60–70	308 (30.17)	1868 (17.02)
	≥70	313 (30.66)	1688 (15.38)
**Sex**	Male	590 (57.79)	6281 (57.22)
	Female	431 (42.21)	4696 (42.78)
**SES**	White collar	430 (42.12)	5121 (46.65)
	Blue collar	345 (33.79)	4125 (37.58)
	Not gainfully employed or missing	246 (24.09)	1731 (15.77)
**BMI**	Mean (SD)	25.03 (4.31)	24.52 (3.21)
	<18.5 kg/m^2^	3 (0.29)	31 (0.28)
	18.5–25 kg/m^2^	105 (10.28)	1624 (14.79)
	25–30 kg/m^2^	65 (6.37)	860 (7.83)
	>30 kg/m^2^	15 (1.47)	125 (1.14)
	Missing	833 (81.59)	8337 (75.95)
**Fasting status**			
1^st^ measurement	Fasting	681 (66.70)	7494 (68.27)
	Non fasting	188 (18.41)	2064 (18.80)
	Missing	152 (14.89)	1419 (12.93)
2^nd^ measurement	Fasting	707 (69.25)	7584 (69.09)
	Non fasting	194 (19.00)	2222 (20.24)
	Missing	120 (11.75)	1171 (10.67)
3^rd^ measurement	Fasting	716 (70.13)	7432 (67.71)
	Non fasting	191 (18.71)	2362 (21.52)
	Missing	114 (11.17)	1183 (10.78)
4^th^ measurement	Fasting	688 (67.38)	7301 (66.51)
	Non fasting	243 (23.80)	2764 (25.18)
	Missing	90 (8.81)	912 (8.31)
**Parity** *	Nulliparity	108 (25.06)	1207 (25.70)
	1	83 (19.26)	928 (19.76)
	2	160 (37.12)	1671 (35.58)
	≥3	80 (18.56)	890 (18.95)
**Age at first childbirth** *	Nulliparity	108 (25.06)	1207 (25.70)
	≤20	44 (10.21)	473 (10.07)
	20–25	114 (26.45)	1348 (28.71)
	25–30	113 (26.22)	1148 (24.45)
	30–35	36 (8.35)	389 (8.28)
	≥35	16 (3.71)	131 (2.79)
**History of cardiovascular disease**		176 (17.24)	1214 (11.06)
**History of lung disease**		66 (6.46)	752 (6.85)
**History of diabetes**		19 (1.86)	128 (1.17)
**Mean follow-up time (years) (SD)**		5.71 (3.34)	9.74 (2.61)
**Triglycerides (mmol/L) –** Overall mean (SD)		1.46 (0.79)	1.39 (0.93)
**Total cholesterol (mmol/L) –** Overall mean (SD)		5.97 (0.93)	5.82 (1.04)
**Albumin (g/L) –** Overall mean (SD)		41.77 (2.17)	42.67 (2.32)
**Glucose (mmol/L)**	Overall mean (SD)	5.38 (1.46)	5.18 (1.38)
1^st^ measurement	Mean (SD)	5.35 (1.75)	5.15 (1.60)
	<5.6 mmol/L	783 (76.69)	9052 (82.46)
	5.6–6.9 mmol/L	165 (16.16)	1288 (11.73)
	≥7 mmol/L	73 (7.15)	637 (5.80)
2^nd^ measurement	Mean (SD)	5.38 (1.81)	5.15 (1.48)
	<5.6 mmol/L	758 (74.24)	8995 (81.94)
	5.6–6.9 mmol/L	177 (17.34)	1372 (12.50)
	≥7 mmol/L	86 (8.42)	610 (5.56)
3^rd^ measurement	Mean (SD)	5.40 (1.55)	5.20 (1.58)
	<5.6 mmol/L	741 (72.58)	8835 (80.49)
	5.6–6.9 mmol/L	197 (19.29)	1460 (13.30)
	≥7 mmol/L	83 (8.13)	682 (6.21)
4^th^ measurement	Mean (SD)	5.40 (1.55)	5.24 (1.60)
	<5.6 mmol/L	756 (74.05)	8717 (79.41)
	5.6–6.9 mmol/L	169 (16.55)	1528 (13.92)
	≥7 mmol/L	96 (9.40)	732 (6.67)
**Fructosamine (mmol/L)**	Overall mean (SD)	2.14 (0.24)	2.13 (0.24)
	Overall mean c-fructosamine (SD)	5.13 (0.59)	5.00 (0.60)
1^st^ measurement	Mean (SD)	2.16 (0.29)	2.15 (0.29)
	Mean c-fructosamine (SD)	5.16 (0.75)	5.04 (0.73)
	≤2.6 mmol/L	969 (94.91)	10459 (95.28)
	>2.6 mmol/L	52 (5.09)	518 (4.72)
2^nd^ measurement	Mean (SD)	2.14 (0.28)	2.13 (0.27)
	Mean c-fructosamine (SD)	5.13 (0.71)	5.00 (0.68)
	≤2.6 mmol/L	968 (94.81)	10538 (96.00)
	>2.6 mmol/L	53 (5.19)	439 (4.00)
3^rd^ measurement	Mean (SD)	2.13 (0.28)	2.12 (0.28)
	Mean c-fructosamine (SD)	5.12 (0.69)	5.00 (0.71)
	≤2.6 mmol/L	965 (94.52)	10503 (95.68)
	>2.6 mmol/L	56 (5.48)	474 (4.32)
4^th^ measurement	Mean (SD)	2.12 (0.28)	2.12 (0.28)
	Mean c-fructosamine (SD)	5.12 (0.69)	4.98 (0.70)
	≤2.6 mmol/L	974 (95.40)	10493 (95.59)
	>2.6 mmol/L	47 (4.60)	484 (4.41)

* Measured in women.

When assessing the link between glucose, fructosamine and overall cancer risk in the first model, we only observed a positive association between standardized log of overall mean glucose and cancer (HR = 1.08; 95% CI: 1.02–1.14). When we included both glucose and fructosamine in one model, we found a stronger association between glucose and cancer risk and a statistically significant inverse association between fructosamine and overall cancer (HR = 1.17; 95% CI: 1.08–1.26 and 0.89; 95% CI: 0.82–0.96 for every standard deviation increase in log of glucose and fructosamine, respectively). Further adjustments for potential confounders did not substantially alter these results (Table 2, nor did including standardized log c-fructosamine instead of fructosamine (results not shown). Furthermore, we performed a sensitivity analysis for persons aged 50 and older and found similar results (HR = 1.11; 95%: 1.02–1.22 and 0.89; 0.81–0.98 for every SD increase in log glucose and fructosamine, respectively; P-value for interaction 0.15).

**Table 2:** Hazard ratios and confidence intervals for the risk of overall and different types of cancer for standardized log overall mean glucose and fructosamine.

	HR (95% CI)				P-value forInteraction*
	Standardized logglucose	P-value	Standardised log fructosamine	P-value	
**Overall cancer**					
Model 1^1^	1.08 (1.02–1.14)	0.008	0.98 (0.93–1.05)	0.62	
Model 2^2^	1.14 (1.05–1.24)	0.002	0.88 (0.81–0.96)	0.003	0.10
Model 3^2,3^	1.14 (0.91–1.44)	0.26	0.93 (0.75–1.17)	0.55	0.29
Adjusted for BMI	1.14 (0.90–1.44)	0.30	0.94 (0.75–1.18)	0.59	0.30
Model 4^2,4^	1.27 (1.10–1.46)	0.001	0.92 (0.83–1.03)	0.13	0.63
Model 5^5^	1.09 (0.97–1.23)	0.06	0.97 (0.86–1.09)	0.23	0.01
**Prostate cancer** 2,7 (N = 220)	1.06 (0.87–1.28)	0.57	0.79 (0.65–0.96)	0.02	0.18
**Breast cancer** 2,8 (N = 137)	1.12 (0.88–1.44)	0.36	0.93 (0.73–1.17)	0.52	0.58
**Colorectal cancer** 2 (N = 128)	1.26 (0.99–1.60)	0.06	0.73 (0.57–0.93)	0.01	0.24
**Lung cancer** 2 (N = 57)	1.32 (0.90–1.93)	0.15	0.54 (0.37–0.79)	0.001	0.96

* Interaction between glucose and fructosamine in relation to cancer risk.

1 Standardized log glucose and fructosamine were each analyzed in separated models; adjusted for age.

2 Adjusted for age, sex, SES, fasting status, history of diabetes, lung and cardiovascular disease, serum albumin, total cholesterol and triglycerides.

3 Subcohort of those with BMI values (N = 2,828).

4 Subcohort of nondiabetic persons, defined as those with serum glucose level <7.0 mmol/L at all measurements and without registered hospital discharge diagnosis of diabetes mellitus prior to the date of last measurement (N = 10,743); not adjusted for history of diabetes.

5 Subcohort of fasting persons; not adjusted for fasting status (N = 5,026);

6 Stratified analysis by glucose tertiles to evaluate the interaction between glucose and fructosamine; standardized log glucose was not included in the model.

7 Sex-stratified analysis in men; not adjusted for sex.

8 Sex-stratified analysis in women; not adjusted for sex; adjusted for parity and age at first childbirth.

**Figure 1:**
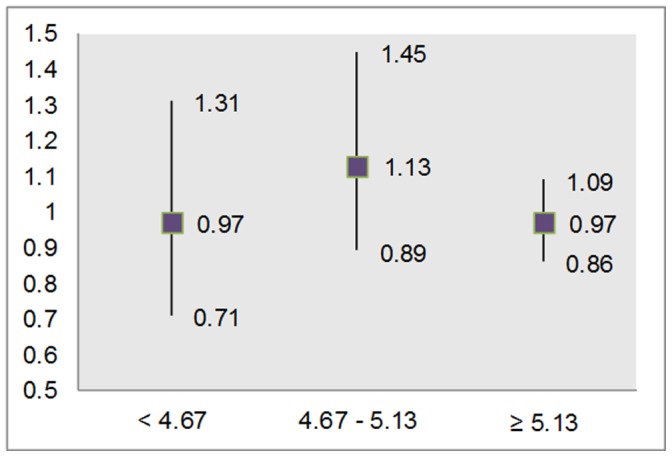
Hazard ratios for overall cancer risk for standardized log fructosamine in different tertiles of glucose in fasting population. The model was adjusted for age, sex, SES, fasting status, history of diabetes, lung and cardiovascular disease, serum albumin, total cholesterol and triglycerides.

In the subgroup of non-diabetic persons, the association between standardized log of overall mean glucose and the risk of cancer was stronger (HR = 1.27; 95% CI: 1.10–1.46), but no statistically significant association between fructosamine and cancer risk was found. In the subgroup with recorded BMI values and the subgroup who was fasting during all four measurements, we did not observe any relationship between glucose, fructosamine, and cancer risk (Table 2). When conducting a test for interaction, we found a statistically significant interaction between glucose and fructosamine in the fasting population. We subsequently performed a stratified analysis of fructosamine by glucose tertiles but no marked difference was seen for the link between fructosamine and cancer in different glucose tertiles (Figure 1).

When studying specific cancers, we observed an inverse association between standardized log of overall mean fructosamine and risk of prostate, colorectal, and lung cancer when also taking into account glucose levels (HR = 0.79; 95% CI: 0.65–0.96, 0.73; 0.57–0.93 and 0.54; 0.37–0.79, respectively). No clear association was found between glucose, frucosamine and breast cancer risk. Finally, using likelihood ratio test, we found no statistically significant interaction between standardized log glucose and fructosamine in the above models (Table 2).

**Figure 2:**
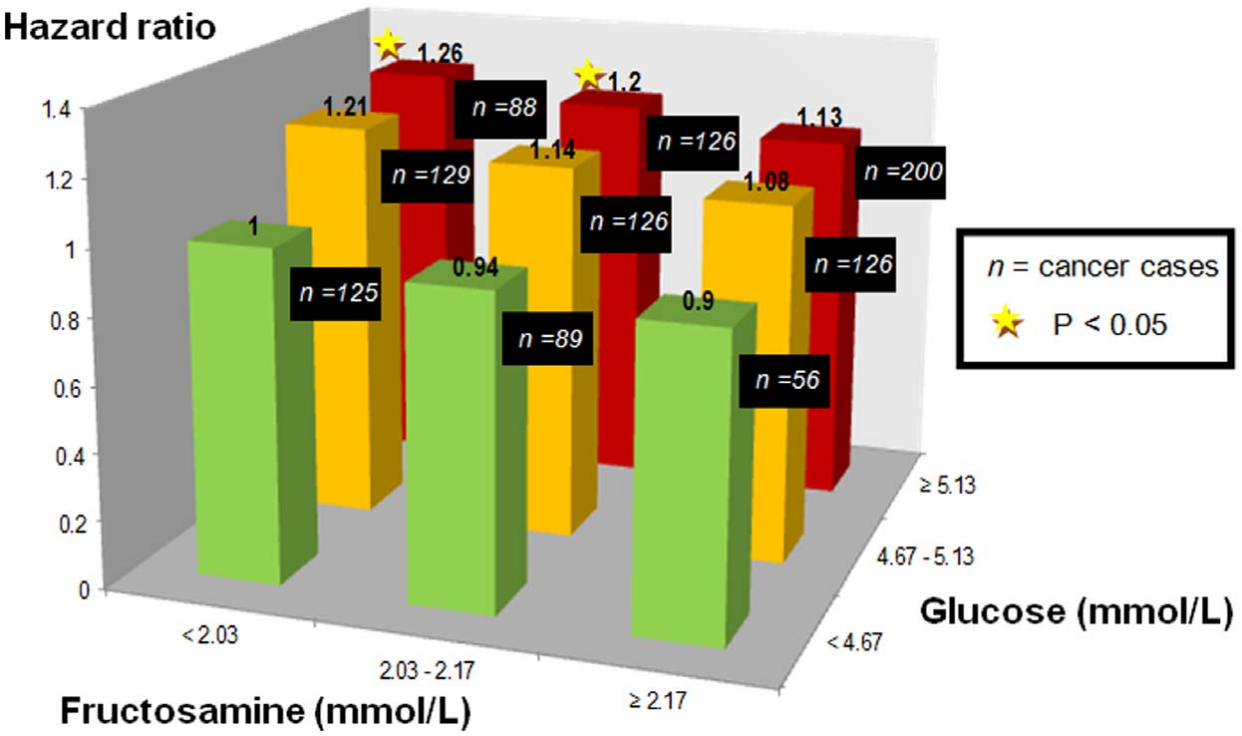
Hazard ratios for overall cancer risk for groups of population based on tertiles of overall mean glucose and fructosamine. All models were adjusted for age, sex, SES, fasting status, history of diabetes, lung and cardiovascular disease, serum albumin, total cholesterol and triglycerides. P-value for interaction was 0.77.

**Figure 3:**
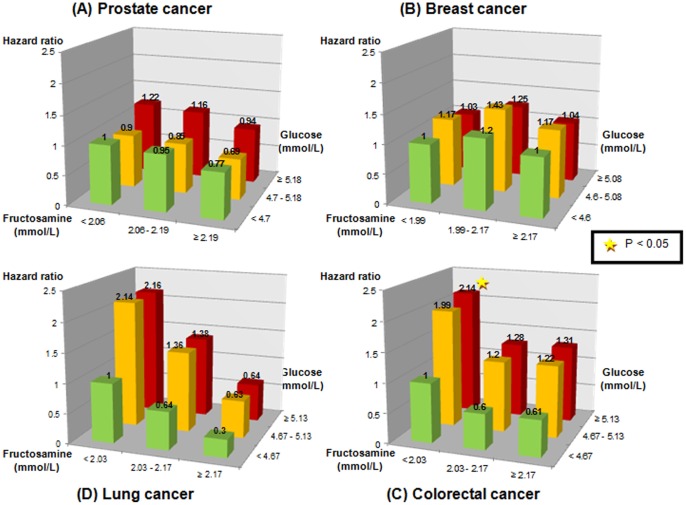
Hazard ratios for the risk of different types of cancer for groups of population based on tertiles of overall mean glucose and fructosamine. All models were adjusted for age, SES, fasting status, history of diabetes, lung and cardiovascular disease, serum albumin, total cholesterol and triglycerides. Additional adjustment for sex was performed for colorectal and lung cancer, as well as for parity and age at first childbirth for breast cancer. P-values for interaction were 0.29, 0.93, 0.01, and 0.08 for prostate, breast, colorectal and lung cancer, respectively.

Figure 2 shows the patterns of cancer risk when looking at combinations of tertiles of glucose and fructosamine. When considering the combination of the lowest tertiles of glucose and fructosamine as the reference, we found an increase in cancer risk for those in higher tertiles of glucose and lower tertiles of fructosamine, although the P-values were not statistically significant. The highest risk of developing cancer was found in those in the highest tertile of glucose and lowest tertile of fructosamine. Similar patterns were seen for risk of prostate, colorectal, and lung cancer (Figure 3).

In order to test whether the subgroup with four repeated measurements differs from those with at least one glucose measurement in AMORIS, we studied this association in the subcohort of all persons aged >20 with a single measurement of glucose and fructosamine (n = 402,026). As shown in Table 3, we found the pattern of association between glucose, fructosamine and cancer risk in this cohort to be similar to the above results (HR for overall cancer risk was 1.09; 95% CI: 1.07–1.20 and 0.93; 95% CI: 0.92–0.94 for every standard deviation increase of log overall mean glucose and fructosamine, respectively). Neverthelesss, more confounding by other factors was found in the population with a single measurement, suggesting a superiority of repeated measurements in reflecting average glycaemic status.

**Table 3:** Comparison between population with repeated measurements and single measurement of glucose and fructosamine in the AMORIS Study.

	Repeated measurements(N = 11,998)	Single measurement(N = 402,026)
Mean age in years (cancer/no cancer)	62.82/52.88	55.87/44.31
Cancer cases (%)	1,021 (8.51%)	27,069 (6.73%)
HR (95%) for overall cancer for every SD increase in log glucose*	1.14 (1.05–1.24)	1.09 (1.07–1.20)
HR (95%) for overall cancer for every SD increase in log fructosamine*	0.88 (0.81–0.96)	0.93 (0.92–0.94)
Confounding factors with statistically significant effect on overallcancer risk	Age, sex	Age, sex, SES, history of diabetes, lung and cardiovascular disease, total cholesterol, tryglicerides, albumin

* Standardized log glucose and fructosamine were each analyzed in the same models; adjusted for age, sex, SES, fasting status, history of diabetes, lung and cardiovascular disease, serum albumin, total cholesterol and triglycerides.

## Discussion

The present study found an increased risk of cancer in persons with increasing glucose levels and decreasing fructosamine levels. We also showed that those in the highest tertile of glucose and lowest tertile of fructosamine combined were at the highest risk of developing cancer. Similar findings were observed for prostate, colorectal, and lung cancer.

The generation of advanced glycation end products (AGEs) during nonenzymatic glycation of free amino group of proteins, lipids, or amino acids is one of the suggested mechanisms for the link between elevated serum glucose and cancer risk [7]. Binding of AGEs to their receptor (RAGE) triggers a series of cellular signaling cascades leading to chronic inflammation, which is thought to contribute to cancer [24]. Additionally, serum glucose enhances the compensatory production of insulin, a strong growth factor, in the early stage of diabetes and in insulin resistant states [25], [26]. Elevated insulin levels have also been linked to increased insulin-like growth factor I (IGF-I), a more potent proliferative and anti-apoptotic agent compared to insulin [27]. Both insulin and IGF-I are therefore thought to explain the potential role of impaired glucose metabolism in carcinogenesis [8], [28].

Fructosamine represents all glycated serum proteins and may therefore also be related to formation of AGEs and subsequent development of cancer [7]. At time of sampling in the present study, fructosamine was widely used in addition to glucose as a suitable integrated measurement of glycaemia, however, HbA_1C_ has later taken over this role and is currently regarded as the gold standard for measurement of glycaemic control in diabetic patients [14]. Few studies have studied fructosamine in the context of cancer. Misciagna et al. reported that elevated fructosamine level (≥2.7 mmol/L) was associated with an increased risk of colorectal adenoma (OR = 2.15; 95% CI: 1.07–4.34) [16]. Platek et al, on the other hand, demonstrated no clear association between fructosamine and breast cancer risk [15]. Meanwhile, other studies linking impaired glucose metabolism and cancer demonstrated a non-linear, U-shaped association between HbA_1C_ and cancer occurrence [29], [30], whereas fasting glucose presented a linear association [30]. The latter is congruent with the present study as higher glucose levels were associated with increased cancer risk as opposed to the inverse association between fructosamine and cancer risk. Additionally, a recent study by Jiao and colleagues showed that a higher risk of colorectal cancer was found in those with higher levels of AGE after adjustment for soluble receptor for AGE (sRAGE) [31], which may underlie the association between the glucose metabolism and cancer via the aforementioned mechanism. No statistically significant trend was observed between sRAGE-adjusted AGE and risk of colorectal and pancreatic cancer [32]. Nevertheless, the same authors suggested that sRAGE, which has AGE-binding capacity without eliciting cellular effects, may lower the risks of these malignancies (31, 32).

When interpreting the value of glucose and fructosamine as biomarkers, one should take into account the significant differences between measures of plasma glucose and glyceamia estimated from HbA_1C_ and fructosamine in turnover times. The 6 to 12 week time frame over which HbA_1C_ equilibrates is important when comparing it with shorter-term measures as fructosamine [33]. Another important consideration in the clinical interpretation of fructosamine concentrations may be an effect of variations in serum protein concentrations [34]. However, the latter was not demonstrable in the present study as most participants had normal values of albumin throughout all measurements and glucose was equally correlated to fructosamine and c-fructosamine. Apart from the temporal factors, the persistent discordances between HbA1c and fructosamine may also be caused by the fact that plasma glucose and fructosamine reflect the physiology of glucose in the extracellular space, whereas HbA_1C_ reflects non-enzymatic glycosylation in the intraerythrocyte compartment [33]. However, the difference in physiologic compartments reflected in fructosamine and HbA_1C_ is beyond the scope of this study.

The differences between fasting glucose, fructosamine and HbA_1C_ identify further possible sources of population variation beyond glycaemic control per se. When diagnosing diabetes, postprandial glucose levels are more likely to detect diabetes in lean individuals, while fasting glucose levels are more likely to identify obese individuals [35]. Thus, obesity may partly be involved in the different associations between glucose, fructosamine, and cancer risk. However, we did not observe any relation between glucose, fructosamine and cancer risk in the subgroup with baseline BMI, which may be caused by the lack of statistical power due to the small number of cancer cases in this subcohort. Further investigation on this subject is necessary to clarify whether glucose and fructosamine identify two not fully overlapping populations with different cancer risks.

To date, there have been few studies on repeated measurements of glucose metabolism markers in relation to cancer risk. Kabat *et al*. assessed the risk of breast and colorectal cancer in the Women’s Health Initiative Study using serum glucose as a time-dependent covariate and demonstrated a positive trend between glucose measured in different time frames and the risk of cancer [12], [13]. However, since reverse causation may exist between impaired glucose metabolism and cancer, the use of glucose as a time-dependent covariate in assessing the risk of cancer must be interpreted with great caution [36].

In this study, an inverse relation between fructosamine and cancer risk was also observed for different cancer sites, i.e prostate, colorectal and lung cancer. However, most of the associations between glucose and risk of these individual cancers were not statistically significant, which may be caused by the small number of events in the study population. Additionally, such lack of association may occur due to other factors modifying individual cancer risk, such as menopausal status and hormone replacement therapy for breast cancer [37], [38]. Also using the AMORIS population, Lambe et al demonstrated a slightly elevated risk of breast cancer in postmenopausal women with impaired glucose metabolism (HR = 1.11; 95% CI: 0.96–1.28) compared to normal glucose levels) which emphasizes the importance of taking hormonal factors into account [39]. For prostate cancer, an increased risk was observed in persons in the third tertile of overall mean glucose, which contradicts prior findings of a protective role of glucose [40]. Following the reduced risk of prostate, colorectal and lung cancer in those with lower fructosamine levels in the present study, a more complex metabolic process may be underlying the relation between the glucose metabolism and development of individual cancers. In addition, as smoking is a major determinant in lung cancer and it may also influence glucose homeostasis [41], the strong inverse association between fructosamine and lung cancer in this study may be confounded by the link between smoking and other lifestyle factors with the glucose metabolism.

The major strength of this study is the large number of subjects with four prospectively collected measurements of serum glucose and fructosamine, all measured in the same laboratory. The use of national registers provided detailed follow-up information on diagnosis of cancer, time of death, and emigration for all subjects. To our knowledge, this is the first study using repeated measurements of glucose and fructosamine in assessing the risk of cancer, hence providing a more detailed and accurate overview of the glucose status of an individual. The AMORIS population was mainly selected by analyzing blood samples from healthy check-ups in non-hospitalized individuals. This healthy cohort effect is not thought to affect the internal validity of the current study and is likely to be minor since it has been shown that the AMORIS cohort is similar to the general working population of Stockholm County in terms of SES and ethnicity [42]. A limitation of this study is that there was no record of outpatient diagnosis of diabetes and diabetes medications. Nonetheless, the models were adjusted for history of diabetes according to hospital discharge diagnosis and a model of subjects without an inpatient diagnosis of diabetes was developed to evaluate the effect caused by the diagnosis of diabetes. There was also limited information regarding obesity, although BMI adjustment did not change our results in the subcohort with baseline BMI. Furthermore, there was no information for other possible confounders such as smoking status and alcohol consumption. However, history of lung disease was used as a proxy for smoking.

### Conclusion

By using repeated measurements of glucose and fructosamine, the present study showed that higher levels of fructosamine contributed to a decreased risk of cancer as opposed to the effect of higher glucose levels on cancer risk. These discrepancies emphasize the complex relation between the glucose metabolism and cancer, which does not necessarily reflect the established link between diabetes and cancer. Instead, our findings highlight the existence of different cancer risk groups among those with impaired glucose metabolism, according to their individual metabolic profiles. Hence, further studies are relevant to understand whether glucose levels are associated with cancer risk as markers of lifestyle factors or metabolic changes other than diabetes rather than as biomarkers of diabetes or insulin resistance.
